# Analysis of peak locomotor demands in women’s football–the influence of different epoch lengths

**DOI:** 10.1371/journal.pone.0303759

**Published:** 2024-05-23

**Authors:** Ivan Baptista, Andreas K. Winther, Dag Johansen, Svein Arne Pettersen

**Affiliations:** 1 Department of Computer Science, Faculty of Science and Technology, UiT The Arctic University of Norway, Tromsø, Norway; 2 Faculty of Sport, Center of Research, Education, Innovation, and Intervention in Sport (CIFI2D), University of Porto, Porto, Portugal; 3 School of Sport Sciences, Faculty of Health Sciences, UiT The Arctic University of Norway, Tromsø, Norway; Portugal Football School, Portuguese Football Federation, PORTUGAL

## Abstract

The quantification of peak locomotor demands has been gathering researchers’ attention in the past years. Regardless of the different methodological approaches used, the most selected epochs are between 1-, 3-, 5- and 15-minutes time windows. However, the selection of these time frames is frequently arbitrary. The aim of this study was to analyse the peak locomotor demands of short time epochs (15, 30, 45, and 60 seconds) in women’s football, with special emphasis over the high-speed metrics. During two seasons, the match physical performance of 100 female football players was collected with Global Positioning System units (STATSports Apex). Peak locomotor demands for the selected variables were calculated by using a 1-second moving average approach. For statistical procedures, linear mixed modelling was used, with total distance, high-speed running distance (>16 km∙h^-1^), sprint distance (>20 km∙h^-1^), and acceleration and deceleration distance (±2.26 m∙s^-2^) considered as the dependent variables and the epoch lengths (15, 30, 45, and 60 seconds) considered as the independent variables. A novel finding was the high ratio observed in the 15 seconds epochs of high-speed running distance and sprint distance (77.6% and 91.3%, respectively). The results show that most peak high-speed demands within 60 seconds are completed within just 15 seconds. Thus, intensity-related variables, such as high-speed metrics, would be better contextualised and adapted into training practices if analysed in shorter epoch lengths (15–30 seconds), while longer periods might be used for volume-related metrics (i.e., total distance), depending on the purpose of the analysis.

## Introduction

The quantification of peak locomotor demands, commonly known as worst-case scenarios, has received researchers’ attention in the past years, and the available literature about this concept is considerably growing [1]. Its relevance within the football domain has previously been shown by several authors [2–4], particularly when direct comparisons with the whole-match average physical demands are made [5]. The applicability of this load monitoring strategy is varied and can be useful for the reduction of injury potential risk, recovery strategies, training interventions, and individualization of the training process [6–8].

Peak locomotor demands have been studied from different perspectives, with research focusing on their temporal distribution [9], methodological approaches [10, 11], and on the influence of certain contextual factors, such as playing-position [12], tactical role [13], match location [14], congestion periods [15], playing formation [16], match status [17], and match outcome [14]. Regardless the different methodological approaches used (fixed or rolling average method) the most selected epochs are 1-, 3-, 5-, and 10-min time windows [7]. However, the rationale for choosing these specific time frames is frequently arbitrary, with researchers seldom considering the applicability of such reference values into training practices [3].

It is consistent in the available literature that higher intensities of peak locomotor demands are observed when the epoch length is shorter [10, 12, 14, 17–21]. For instance, considerably different intensities of high-speed running distances were reported between 1-, 2-, and 3-min peaks (54.3, 32.6, and 25.0 m/min, respectively) [10]. García et al. [21] compared five different time epochs (30, 60, 120, 180, and 300s) across five different team sports and interesting results were found, particularly between the 30s peaks and the 60s peaks. Despite the expected higher intensity of the 30s peak, the fact that this period represents >87% of the distance covered at high speed in the 60s peak period (63.8 vs. 72.8 m) is indicative of the remaining challenges researchers and practitioners face when analysing only time epochs over 1 min. Indeed, even in 1-min peak periods, low-intensity activities, such as standing, walking, or jogging, might be highly represented, and studies in other modalities have already suggested further investigation of the peak locomotor demands in shorter time-epochs (<1 min) [22].

Additionally, previous research [23] suggests that the relative intensity (m/min) obtained from a 3-min peak analysis in high-speed variables seems too low to induce changes in the players’ fitness capacity. Different duration-specific intensities may be used with different priorities, with short time windows (<1 min) being more appropriate for running conditioning drills (i.e. sprint training and speed endurance training) if repeated over time, while longer periods (e.g., 5 to 10 min) could be useful for more football-specific training drills, where other factors of performance (technical and tactical) might be replicated [4].

Two recent systematic reviews summarized the data on peak match demands in football [4, 7] and revealed a bias towards research in male athletes. A total of only three studies investigating female match-play were reported [24–26], and even though few other studies exist [27, 28], none of them have attempted to investigate epoch lengths <1 min. Women soccer players’ performance during the peak locomotor demands are also, as it occurs with men, expected to vary according to the epoch length used. However, major fitness differences between the sexes have previously been shown [29], with the largest disparities being evident in high-speed variables [30].

Furthermore, a recurrent limitation present in this type of research, and where the few studies on women’s football are included, is the use of a single team, which compromises the generalizability of the findings reported [4]. Therefore, the present study aimed to quantify and compare the peak locomotor demands of very short time epochs (15s, 30s, 45s, and 60s) in multiple female professional football teams, with special emphasis over the high-speed metrics.

## Methods

Before initiating the study, ethical approval was sought from the Regional Committee for Medical and Health Research Ethics–Northern Norway (reference number 53884). However, we were exempted as the data collection did not involve a biobank, medical, or health data related to illness, nor did it disrupt the normal operation of the players. Following approval from the Norwegian Centre for Research Data (reference number: 296155), written informed consent was obtained from 100 female football players (22.3 ± 3.7 years of age) representing four teams in the Norwegian premier division. These players were classified as highly trained according to the criteria outlined by McKay et al. [31]. Goalkeepers were not included in this study. From March 2020, we conducted a prospective observational study, collecting tracking data from a total of 153 official matches spanning two full seasons using STATSports Apex (Newry, Northern Ireland), with a sampling frequency of 10 Hz. The tracking system’s validity and accuracy (bias = 1–2%) have been presented elsewhere [32]. All home matches were played on artificial grass, with only occasional away games on natural grass. Players wore their Global Positioning System (GPS) unit on their upper back during matches, adhering to manufacturer instructions. To minimize inter-device error each player consistently used the same GPS unit throughout data collection [32].

Adhering to GPS reporting standards [33], raw GPS data from the manufacturer’s software (STATSports Sonra 2.1.4) were exported into a Python (3.9.12) script for pre-processing. The pre-processing process consisted of applying a 1-second moving average to the raw data signal before deriving distance and acceleration from this smoothed signal. Subsequently, signals for total distance (TotDist), high-speed running distance (HSRD) (>16 km∙h^-1^), sprint distance (SpD) (>20 km∙h^-1^), and acceleration and deceleration distance (Acc_dist_, Dec_dist_) were generated by only keeping distance observations meeting the thresholds listed for each metric. After the pre-processing, a 15-, 30-, 45- and 60-second rolling rum was applied to each metric. The peak value for each combination of epoch length and metric was then selected as the peak period.

Despite no methodological standardization in the literature of velocity thresholds used in women’s football, and because applying thresholds used in men’s football may result in skewed observations, we opted to follow the proposal of Bradley and Vescovi [34], which was also adopted by other research [35, 36]. Acc_dist_ and Dec_dist_ were defined as the distance covered with a positive or negative change in speed of more than ± 2.26 m∙s^-2^ finishing when the rate of Acc_dist_/Dec_dist_ reached 0 m∙s^-2^.

Instead of setting an arbitrary cut-off and thereby excluding several observations, the minutes played were controlled in the statistical models. The final number of observations ranged from 1615–1650 observations, depending on the epoch length and metric analysed. Upon deriving all the metrics, the data was transferred to an R 4.0.5 [37] script for statistical analysis. Anomalies outside Tukey’s fences criterion (1.5 IQR beyond the 1^st^ and 3^rd^ quartiles) [38] were removed based on dataset inspection and visual examination of histograms and boxplots. For each metric, a linear mixed model was created using the “lme” function in the package “nlme” [39], with TotDist, HSRD, SpD, Acc_dist_, and Dec_dist_ as the dependent variables, and the epoch lengths (15s, 30s, 45s and 60s) and “minutes played” as the independent variables. We also added “player ID”, “team ID”, and “match ID” as a random effects and handled heteroscedasticity by specifying “weigths = varIdent(epochLength)” due to residual variance increasing with epoch duration. Residuals were checked for normality using both residual and QQplot. We then generated estimated marginal means using the package “emmeans” [40] with the Sidak correction for post-hoc comparisons. All results are means with ± 95% confidence intervals unless otherwise stated. Following the journal requirements, all the data underlying the findings of this study are available as supporting information (S1–S4 Files).

## Results

**Fig 1:**
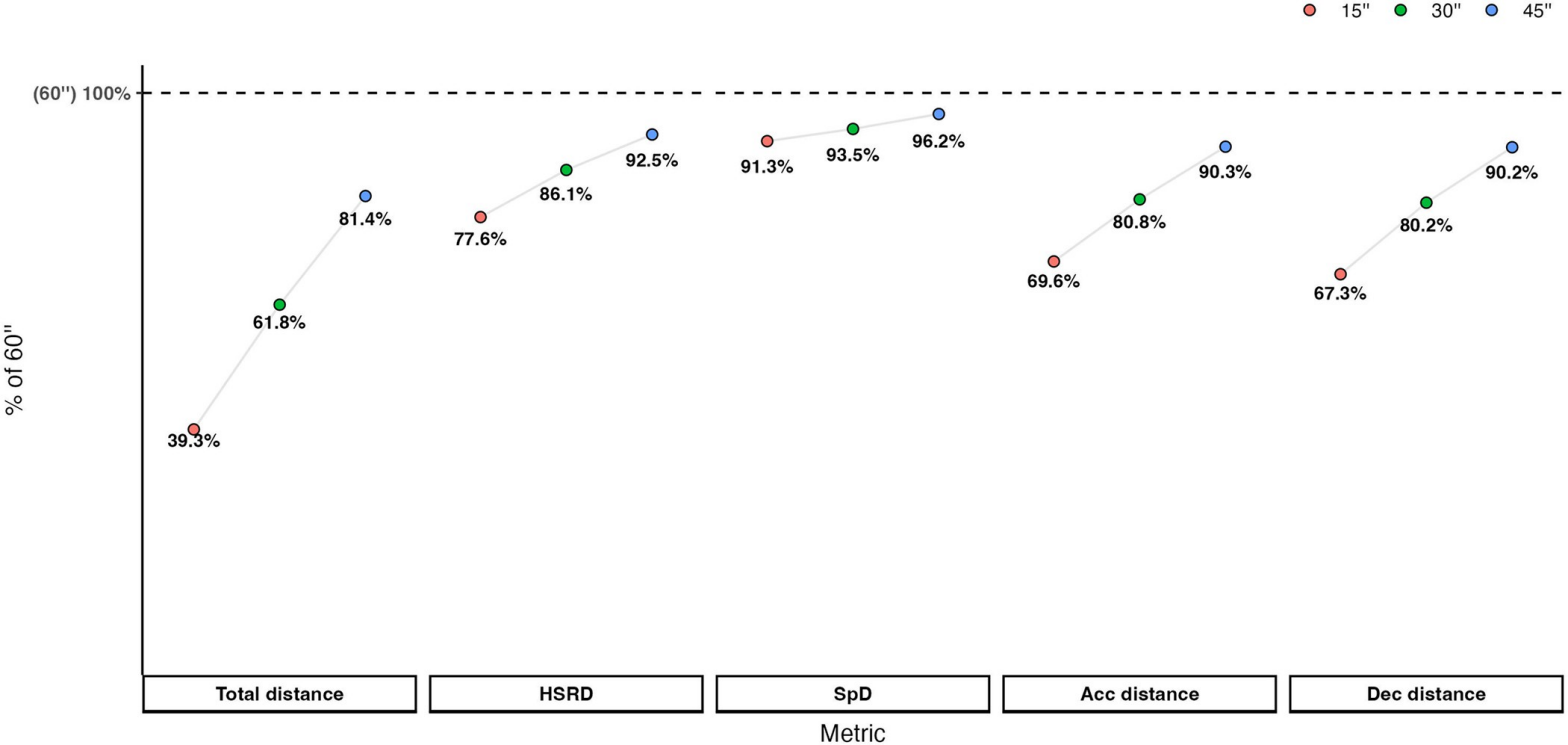
Peak locomotor demands of 15-, 30- and 45s epoch lengths in percentage of 60s peak periods.

General match physical characteristics of the selected players were previously described by Winther et al. [36], and descriptive statistics of the peak locomotor demands for each selected variable across the four different epoch lengths are presented in Table 1. TotDist was the physical performance variable where the highest difference between the 15s (72.4 m) and 60s (182.6 m) periods was observed, while the lowest difference was presented in SpD (38.4 m vs. 41.9 m). The values observed in Acc_dist_ and Dec_dist_ were consistently comparable across all analysed epoch lengths. In Fig 1, the 15-, 30-, and 45s peak locomotor demands are expressed as a ratio of the respective 60s peak period. The highest ratios of the 15-, 30-, and 45s periods were observed in SpD, with percentages of 91.3%, 93.5%, and 96.2%, respectively. While HSRD also presented high ratios in each of these periods (77.6%, 86.1%, and 92.5%), TotDist showed a completely different profile, with the lowest ratios among the physical variables analysed (39.3%, 61.8%, and 81.4%). Acc_dist_ and Dec_dist_ presented ratios of ~70%, ~80%, and ~90% in the 15-, 30-, and 45s peak periods, respectively.

**Table 1:** Peak locomotor demands for the selected variables expressed in meters (means ± SD), across four different epoch lengths.

Epoch length (s)	Total Distance	HSRD	Sprint distance	Acc distance	Dec distance
15	72.4 ± 0.8	55.2 ± 1.0	38.4 ± 0.9	26.1 ± 0.4	22.7 ± 0.3
30	113.2 ± 0.8	60.9 ± 1.0	39.3 ± 0.9	30.2 ± 0.4	26.9 ± 0.3
45	148.9 ± 0.8	65.3 ± 1.0	40.4 ± 0.9	33.6 ± 0.4	30.1 ± 0.3
60	182.6 ± 0.8	70.4 ± 1.0	41.9 ± 0.9	37.1 ± 0.4	33.3 ± 0.3

Total-, HSRD-, (High-speed running distance), Sprint-, Acc-, (Acceleration), Dec- (Deceleration) distance in meters.

## Discussion

This study is the first that, by using a sample from multiple professional female football clubs, objectively reveals the proportions of how the 60s peak locomotor demands are distributed across smaller periods of 15s, 30s, and 45s. The main and novel finding was the high ratio observed in the 15s epochs of HSRD and SpD (77.6% and 91.3%, respectively). These results show that the increase in distance covered at higher thresholds is very small from 15s and onwards. Particularly for SpD, it shows that the remaining 45s are responsible for only 8–9% of the total 60s peak demand, raising the question regarding the utility of using 1-min epoch lengths when analysing peak locomotor demands of high-speed variables. If practitioners use the match intensity observed in the 1-min peak of SpD for training purposes, it means they will replicate relative intensities of ~0.70 m/s. However, if 15s epoch lengths are used (as suggested in the present study), a more than three times higher intensity (~2.56 m/s) will be imposed on the players. For instance, a speed training program based on the ratios obtained through 1-min epochs will not meet the requirements of 15s epochs, since shorter bouts or longer recovery periods may be prescribed when longer epoch lengths are analysed. In this regard, to prepare players to cope with the demands of 15s peaks, the players may need to follow the speed endurance training principles [41]. Conversely, to reproduce the intensities of 1-min peaks, the ‘traditional’ speed training programmes (short sprints interspersed by complete recovery periods [42]) might be enough. This is very relevant insight for practical performance development purposes, since much smaller epoch lengths than 60s resemble more accurately the specific match activity observed.

We, therefore, support the suggestion of Novak et al. [23] regarding the necessity of using shorter epoch lengths if changes in the players’ sprinting and speed endurance capacities are to be induced. Although previous research proposed training drills durations from 1 to 10 min when preparing players to cope with matches’ high-intensity periods [43], we argue that shorter epochs must also be considered during training drills that request high-speed displacements. Furthermore, football matches present a stochastic nature, where bouts of high-intensity efforts are interspersed by low-intensity periods [44]. Previous research [45] suggested that these high-intensity periods coincide with the transitional periods of the match (the moments when the team wins or loses the possession of the ball). These transitions have also been shown to last a maximum of ~20s with a mean duration of around 10s [45, 46], which together with the findings of the present study suggests that consecutive high-speed actions (peak locomotor demands) most likely occur within a specific and short timeframe (transitional periods up to ~20s), followed by a longer period of low or moderate intensity.

While in SpD the increments between the 15-, 30-, 45-, and 60s epochs were 2–4%, slightly higher values were observed for Acc_dist_ and Dec_dist_, with 9–13% increments. However, the metric most distinguished from the others was TotDist, where 19–23% increments were observed between epoch lengths. These results suggest that the duration of the peak locomotor demands should depend on the criterion variable (or group of variables) analysed. Thus, intensity-related variables, such as high-speed metrics (e.g., SpD), would be better contextualised and adapted into training practices if analysed in very short epoch lengths (15-30s). In contrast, longer periods might be used for volume-related metrics (i.e., TotDist), depending on the purpose of the analysis.

More recently, a novel method (ball in play) for analysing the peak locomotor demands has been proposed [16], with the authors suggesting that previous methods (fixed or rolling averages) included game interruptions (i.e., ball out of play). However, the results from their study revealed lower values of high-speed measures in the ball-in-play peak than in the 1-min peak (rolling average), demonstrating that players’ movements and displacements when the ball is out of play should also be accounted for when measuring peak demands. Thus, we suggest that rolling averages should continue to be the preferred method for analysing peak locomotor demands.

The results observed in this study support the rationale for the non-generalisation of the conclusions obtained in men’s football research into women’s football practices. The 1-min peak values of TotDist (182.6 m) were slightly lower than the values previously reported in men’s teams (186 - 201m) [18, 47]. In addition, when comparing the variables of HSRD and SpD, the highest values are observed in our research with women. However, these comparisons must be interpreted cautiously since different speed thresholds are commonly used in men’s and women’s football research. For instance, when comparing variables with similar speed thresholds, we observed that in our study the 1-min peak SpD (41.9 m covered at >20 km∙h^-1^) is considerably lower than the 1-min peak HSRD (~60 m covered at > 19.8 km∙h^-1^) reported in research with male football players [47–49]. Conversely, the study of García et al. [21] observed a ratio (87%) between 30s and 60s epochs in high-speed distance (>18 km∙h^-1^) similar to the ratios reported in our study for HSRD and SpD (86,1% and 93,5%).

Overall, the higher intensities observed in the shorter periods were already expected and followed the same rationale of the findings from Augusto et al. [17] regarding the comparison between 1, 3 and 5-min epoch lengths. However, the varying ratios identified among intensity- and volume-related variables across the analysed epochs, coupled with the consistent high-speed distances recorded during 15s and 60s peak periods, provide insights into the potential application of such information in a practical training context. This underscores the necessity for additional research in order to comprehensively explore and elucidate the implications of these findings.

### Limitations and further research

In the present study, only univariate peak locomotor demands were considered. Despite agreeing with other researchers [12] when mentioning that using a single criterion variable for training tasks designs may limit specificity and underestimate the true peak locomotor demands, the purpose of the present study was to analyse specifically each of the selected variables across the different time epochs. However, further research may add valuable insights to the literature if it manages to analyse and contextualise multivariate peak periods from time epochs <1 min. Another possible limitation of our study is that playing positions were not considered for data analysis. This option was made since previous research has already reported no significant differences between playing positions for HSRD and SpD during time epochs of 1 min, suggesting that high-speed thresholds may limit the appearance of positional differences in peak locomotor demands during match play [12, 14]. However, the inexistence of differences between playing positions is not guaranteed, and further research may find novel results in this regard.

Absolute thresholds were used among the selected physical variables. Despite acknowledging the importance of individualised thresholds, in applied research (real world), it is not always possible to evaluate players’ capacities in order to use individualised high-speed metrics [50].

## Supporting information

S1 File Dataset. (XLSX)

S2 File Means. (XLSX)

S3 File Contrasts. (XLSX)

S4 File Ratios. (XLSX)
